# Recruitment of a SAP18-HDAC1 Complex into HIV-1 Virions and Its Requirement for Viral Replication

**DOI:** 10.1371/journal.ppat.1000463

**Published:** 2009-06-05

**Authors:** Masha Sorin, Jennifer Cano, Supratik Das, Sheeba Mathew, Xuhong Wu, Kelvin P. Davies, Xuanling Shi, S.-W. Grace Cheng, David Ott, Ganjam V. Kalpana

**Affiliations:** 1 Department of Genetics, Albert Einstein College of Medicine, Bronx, New York, United States of America; 2 Department of Microbiology and Immunology, Albert Einstein College of Medicine, Bronx, New York, United States of America; 3 Basic Research Program, SAIC–Frederick Inc., National Cancer Institute–Frederick, Frederick, Maryland, United States of America; Northwestern University, United States of America

## Abstract

HIV-1 integrase (IN) is a virally encoded protein required for integration of viral cDNA into host chromosomes. INI1/hSNF5 is a component of the SWI/SNF complex that interacts with HIV-1 IN, is selectively incorporated into HIV-1 (but not other retroviral) virions, and modulates multiple steps, including particle production and infectivity. To gain further insight into the role of INI1 in HIV-1 replication, we screened for INI1-interacting proteins using the yeast two-hybrid system. We found that SAP18 (Sin3a associated protein 18 kD), a component of the Sin3a-HDAC1 complex, directly binds to INI1 in yeast, *in vitro* and *in vivo*. Interestingly, we found that IN also binds to SAP18 *in vitro* and *in vivo*. SAP18 and components of a Sin3A-HDAC1 complex were specifically incorporated into HIV-1 (but not SIV and HTLV-1) virions in an HIV-1 IN–dependent manner. Using a fluorescence-based assay, we found that HIV-1 (but not SIV) virion preparations harbour significant deacetylase activity, indicating the specific recruitment of catalytically active HDAC into the virions. To determine the requirement of virion-associated HDAC1 to HIV-1 replication, an inactive, transdominant negative mutant of HDAC1 (HDAC1^H141A^) was utilized. Incorporation of HDAC1^H141A^ decreased the virion-associated histone deacetylase activity. Furthermore, incorporation of HDAC1^H141A^ decreased the infectivity of HIV-1 (but not SIV) virions. The block in infectivity due to virion-associated HDAC1^H141A^ occurred specifically at the early reverse transcription stage, while entry of the virions was unaffected. RNA-interference mediated knock-down of HDAC1 in producer cells resulted in decreased virion-associated HDAC1 activity and a reduction in infectivity of these virions. These studies indicate that HIV-1 IN and INI1/hSNF5 bind SAP18 and selectively recruit components of Sin3a-HDAC1 complex into HIV-1 virions. Furthermore, HIV-1 virion-associated HDAC1 is required for efficient early post-entry events, indicating a novel role for HDAC1 during HIV-1 replication.

## Author Summary

The interaction between the host and HIV-1 virus is a dynamic process. While some host cellular proteins mount antiviral response, HIV-1 utilizes other host proteins for its propagation. It is crucial to understand the role of host proteins in HIV-1 replication for successful development of strategies to combat AIDS. INI1/hSNF5 is a cellular protein that directly binds to an HIV-1 protein, integrase (IN). It is selectively encapsidated into HIV-1 virions and is required for the subsequent viral replication. However, how INI1 mediates its effects is not completely understood. Here we report a novel finding that INI1 as well as IN directly binds to SAP18 (Sin3a-associated protein of 18 kD), a component of the cellular Sin3a/HDAC (Histone Deacetylase) complex. The HDAC complexes are multiprotein epigenetic modifiers known to affect cellular transcription. In this report we find that along with INI1, SAP18 and the components of the Sin3a-HDAC1 complex are specifically recruited into HIV-1 virions. In addition, we find that virion-associated deacetylase activity is required for efficient reverse transcription in target cells. These studies indicate that HIV-1 IN protein may recruit HDAC1 complex into the virions for utilizing them in the subsequent stages of replication. Our findings provide new insight into the roles of cellular HDAC1 complex in HIV-1 replication and provide a novel paradigm to understand host-virus dynamic interaction.

## Introduction

HIV-1 replication is characterized by a dynamic interplay between the host and the virus [1]. While host restriction factors inhibit HIV-1 replication at various steps, HIV-1 actively engages cellular proteins for its own replication and for subversion of antiviral effects. Several host factors that either restrict or promote HIV-1 replication have been identified in recent years. A high-throughput screening of siRNA mediated knock-down of cellular proteins identified several hundred host proteins that played activating or inhibitory roles in HIV-1 replication highlighting the importance of studying host-virus interaction during HIV-1 replication [2]. While the high through-put screen allows the identification of proteins whose loss of function is not lethal to cells, it is likely that many host factors that are both essential for cellular function and play a role in HIV-1 replication were not identified in such a screen. Several host factors including INI1/hSNF5 and LEDGF, both of which directly bind to HIV-1 integrase (IN), a virally encoded protein required for insertion viral cDNA into host chromosomal DNA, have been identified using the yeast two hybrid system [3],[4]. LEDGF appears to be involved in tethering integrase to transcriptionally active regions [5]. However, INI1/hSNF5 appears to play dual roles in HIV-1 replication in that while INI1/hSNF5 present in the producer cells appears to be required for HIV-1 replication, INI1/hSNF5 present in the target cells may be inhibitory. Our previous studies have indicated that ectopic (cytoplasmic) expression of a dominant negative mutant of INI1, harboring the minimal IN-binding domain, inhibits HIV-1 p24 release in a manner dependent on IN-INI1 interactions [6]. INI1/hSNF5 is specifically incorporated into HIV-1 virions and the virion associated INI1 is required for early events of HIV-1 replication [1],[6],[7]. Furthermore, cellular INI1 binds to Tat and appears to be required for Tat-mediated transactivation [8]–[11]. Knock-down studies suggested that INI1 is antiviral and inhibits early events [12]. One possibility is that cellular INI1 may play a different role than the virally incorporated INI1 explaining the observed effects on HIIV-1 replication.

The complex role of INI1 during HIV-1 replication necessitates deciphering of its cellular function. INI1/hSNF5 also known as BAF47 or SMARCB1, is a component of the human chromatin remodelling SWI/SNF complex [13]. SWI/SNF is an evolutionarily conserved, multi-subunit, high molecular weight (>2 MDa) complex that remodels chromatin in an ATP-dependent manner. The SWI/SNF complex consists of at least nine subunits that are conserved among eukaryotes [14]. Among these are four core subunits that are required for chromatin remodelling, including the key ATPase subunit (BRG1, BRM, SWI2/SNF2), INI1/hSNF5, BAF170 and BAF155. There are two functionally distinct classes of SWI/SNF complexes in mammalian cells, hSWI/SNF-A or BAF and hSWI/SNF-B or PBAF, and three additional complexes that consist of a mixture of components derived from HDAC (histone deacetylase complex) and SWI/SNF including hBRM, hBRG1 (I) and hBRG1 (II) complexes [14]. It is important to note that INI1/hSNF5 is present in each of these SWI/SNF complexes, suggesting multiple functions for this protein in mammalian cells. The stoichiometry, sub nuclear distribution and exact functions of SWI/SNF complexes have yet to be clearly defined in mammalian cells [14]. INI1 is also a tumour suppressor biallelically deleted in highly malignant paediatric tumours known as rhabdoid tumours [15]. We have found that INI1 causes G0–G1 arrest, and represses *Cyclin D1* transcription by directly recruiting HDAC1 (histone deacetylase) to its promoter [16]. These studies suggest that INI1 can mediate both repression and activation of cellular transcription. But the exact mechanism by which INI1 recruits HDAC1 to the promoters to mediate transcriptional repression is unknown.

Here we report that INI1 and IN directly associate with SAP18, a component of the Sin3a-HDAC1 complex. We report a surprising finding that components of Sin3a-HDAC1, but not the core components of the SWI/SNF complex are specifically incorporated into HIV-1 virions. Furthermore, we found that incorporation of a dominant negative mutant of HDAC1 decreases the HDAC1 activity associated with the HIV-1 virions and that this decrease in HDAC1 activity is correlated to a decrease in infectivity of these virions. Finally, we demonstrate that HDAC1 activity is required for modulating a post-entry step at or before reverse transcription during HIV-1 replication. These results indicate an unanticipated role of IN and INI1 in recruiting the HDAC1 complex, independent of SWI/SNF complex, into HIV-1 virions and provide new insights into the role of INI1/hSNF5 and Sin3a-HDAC1 complex in HIV-1 replication.

## Results

### INI1 directly interacts with SAP18, a component of the Sin3a-HDAC1 complex

**Figure 1:**
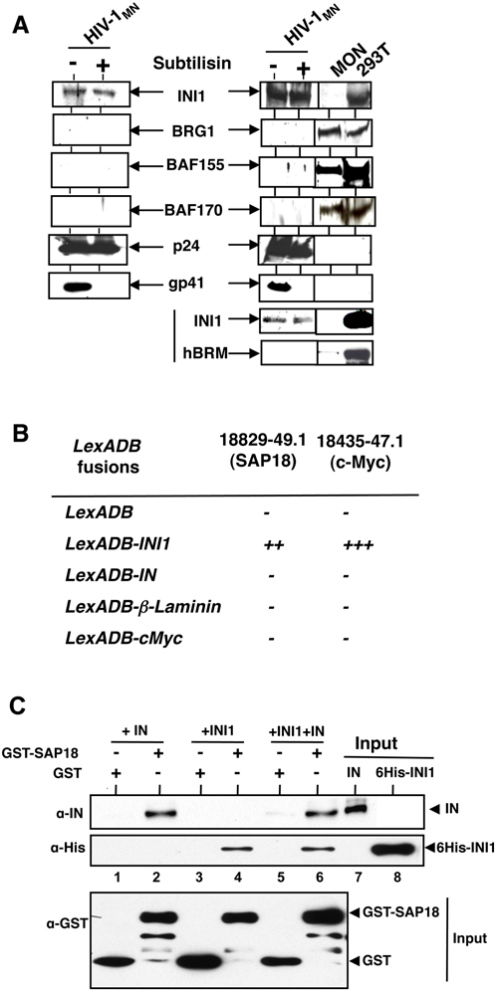
INI1/hSNF5 is incorporated into HIV-1 virions independent of SWI/SNF complex, and directly interacts with SAP18. (A) Purified and concentrated HIV-1_MN_ virions were immunoblotted and the same blot was sequentially probed with α-INI1, α-BRG1, α-BAF155, α-BAF170, α-p24 and α-gp41, and α-hBRM antibodies, as indicated. Lysates from MON (*INI1*−/− rhabdoid cells) and 293T (*INI1*+/+) cells are used as controls. (B) Interaction of SAP18 and INI1 in the yeast two-hybrid system. “++” = Medium interaction; “+++” = strong interaction; and “−” = no interaction, LexADB = LexA DNA binding domain. (C) GST pull-down assay to demonstrate interaction between SAP18, INI1 and IN. Glutathione sepharose beads bound to GST alone or GST-SAP18 were incubated with purified IN or INI1 or both and bound proteins were analyzed by Western blot using the indicated antibodies.

Since INI1/hSNF5 is specifically incorporated into HIV-1 virions [6],[7], we tested to determine if other core components of SWI/SNF complex such as BRG1, BAF155, and BAF170, were present in the virus particles. HIV-1 virions were purified by density gradient centrifugation to separate the microvescicular fraction and the virions were subjected to subtilisin treatment to remove any cellular proteins non-specifically associated with the virions [17]. These purified, subtilisin-treated virions were subjected to immunoblot analysis using antibodies to components of SWI/SNF complex. Protein extracts from: (i) MON (*INI1*−/− rhabdoid cells) that do not express INI1 but express all other components of the SWI/SNF complex; and (ii) 293T cells that express endogenous levels of INI1 and all the components of SWI/SNF complex, were used as controls in these experiments. The same blot was subjected to sequential immunoblot analysis using several different antibodies, to allow the identification of multiple proteins in the same virion preparation. This analysis revealed the presence of INI1, consistent with the previous reports [7]. However, other components of the SWI/SNF complex including BRG1, BRM, BAF155, and BAF170 were not detected, despite the presence of these components in the control 293T cells used as producer cells (Figure 1A and Figure S1). Preliminary density gradient analysis of total nuclear extracts demonstrated that INI1 indeed is present in several high molecular weight fractions, including those devoid of BRG1 (data not shown), suggesting the existence of other unidentified INI1-associated complexes in the cell.

To determine if INI1 could associate with proteins other than SWI/SNF complex, we carried out a yeast two-hybrid analysis where LexADB (DNA Binding domain)-fusion of INI1 was used as bait to screen a HL60 cDNA library fused to GAL4AC (activation domain) [18]. One of the positive clones (#18829-49.1) that specifically interacted with INI1 but not with controls such as LexADB, LexADB-IN, LexaDB-cMYC, LexADB-Laminin and LexADB-cMYC (Figure 1B) was identified as a fragment of SAP18 (aa 45–153). Both cMYC and IN are known INI1 interacting proteins and Laminin is a negative control [18].

### IN and INI1/SNF5 directly interact with SAP18 *in vitro*

To determine if the interaction of SAP18 with INI1 is direct, we carried out a GST pull down assay using purified components of GST (glutathione-S-transferase)-SAP18 and 6H(hexa-histidine)-INI1/hSNF5 fusion proteins, as well as IN. We found INI1 interacts with GST-SAP18 but not GST (Figure 1C, lanes 3 and 4). Interestingly, IN also interacted with GST-SAP18 but not with GST in these assays (Figure 1C, lanes 1 and 2). It is likely that the physical constraints, perhaps present in yeast two hybrid system, may be absent *in vitro*, allowing for the free interaction between IN and SAP18. Furthermore, both IN and INI1 were pulled down by GST-SAP18 suggesting that the three proteins have the potential to form ternary complex (Figure 1C, lanes 5 and 6).

### IN and INI1/hSNF5 co-immunoprecipitate with SAP18 and associate with components of Sin3a-HDAC1 complex *in vivo*

**Figure 2:**
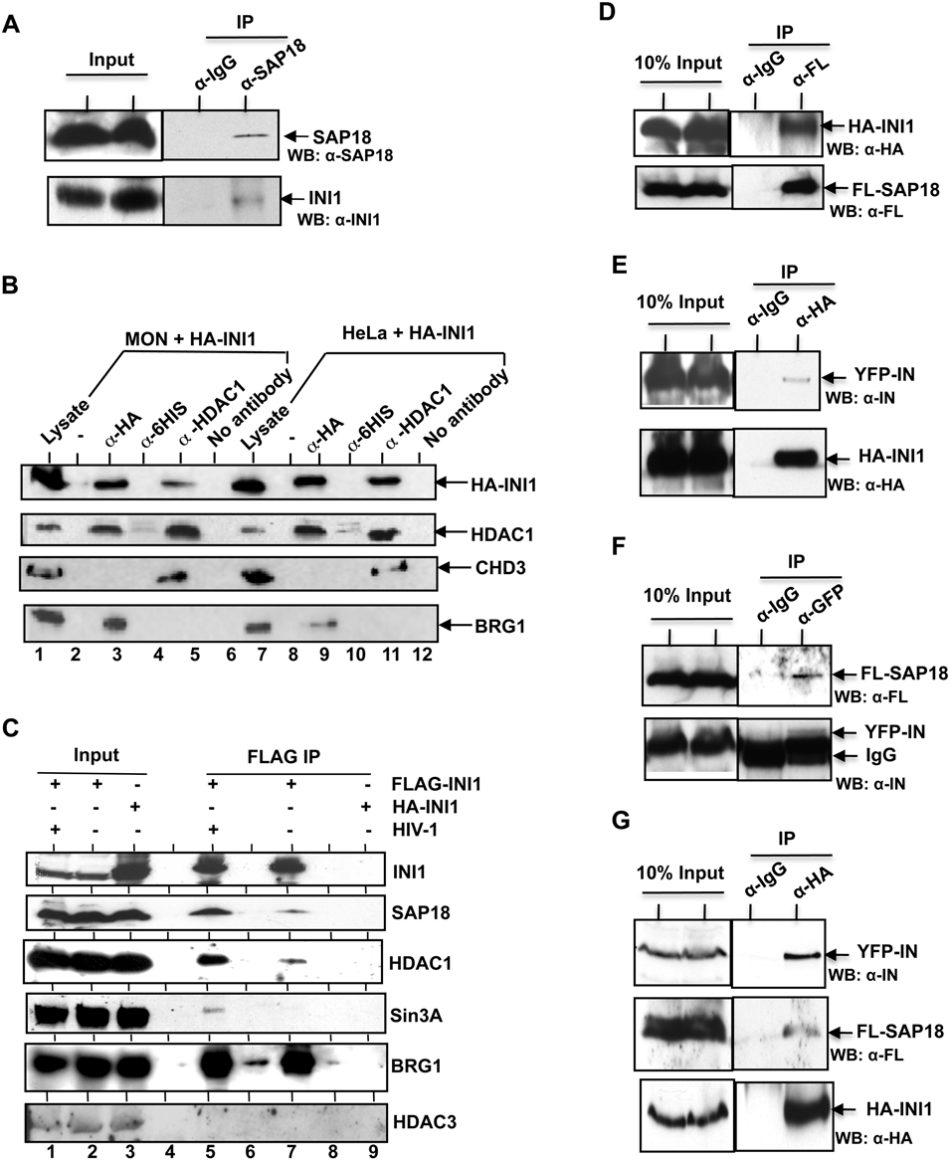
INI1 associates with components of Sin3a-HDAC1 complex in vivo. (A) Interaction between endogenous INI1 and SAP18. Cell lysates from confluent HeLa cells were subjected to co-immunoprecipitation analysis using α-SAP18 antibodies. Rabbit IgG was used as a control. (B) Immunoprecipitation of HA-INI1 with HDAC1 in vivo. Total proteins from MON (INI1−/−) and HeLa cells, transfected with HA-INI1, were subjected to co-immunoprecipitation analyses using α-HA and α-HDAC1 antibodies. No antibodies (beads) and α-6His antibodies were used as negative controls. The immunoblots were sequentially probed with α-HA, α-HDAC1, α-BRG1, and α-CHD3 antibodies. (C) Association of INI1/hSNF5 with components of SIN3a/HDAC1 complex in the presence and absence of HIV-1 viral proteins. 293T cells were co-transfected with HIV-1 viral vectors or an empty vector and plasmid expressing either FLAG-INI1 or HA-INI1. Immunoprecipitation was performed using α-FLAG antibodies. Immunoblots were sequentially probed with α-INI1, α-SAP18, α-HDAC1, α-SIN3A, α-BRG1 and α-HDAC3 antibodies as indicated. (Note: A weak Sin3a band is visible upon longer exposure in lane 7. The BRG1 band in the lane 6 is due to spill over from lane 5). (D–G) In vivo association of HA-INI1, GFP-IN, and FLAG-SAP18. 293T cells were transfected with vectors expressing FL-SAP18 and HA-INI1 (D), HA-INI1 and YFP-IN (E), FL-SAP18 and YFP-IN (F), or FLAG-SAP18, HA-INI1, and YFP-IN (G). Immunoprecipitations were carried out using α-FLAG (D), α-HA (E), α-GFP (F), or α-HA (G) antibodies. Bound proteins and input controls were immunoblotted with α-HA, α-FLAG, or α-IN antibodies. In all panels, WB = Western blot analysis.

To further corroborate the *in vitro* finding, we carried out a series of co-immunoprecipitation assays. First, we found that antibodies to SAP18 could co-immunoprecipitate endogenous INI1, indicating that the two endogenous proteins interact with each other *in vivo* (Figure 2A).

HDAC1 is associated with several complexes, such as Sin3a, NuRD and CoREST [19]. Both Sin3a and SAP18 are components of Sin3a-HDAC1 complex but not that of NuRD and CoREST complexes. On the other hand, MTA1 and CHD3 are exclusive components of NuRD complex but not that of Sin3a complex [19]. To determine if INI1 can specifically associate with the Sin3a-HDAC1 complex, we carried out immunoprecipitation studies to determine the ability of INI1 to pull down HDAC1 and CHD3. MON (INI1−/− rhabdoid cells) and HeLa cells were transfected with a plasmid expressing HA-INI1/hSNF5, and total proteins were immunoprecipitated with either α-HA, α-HDAC1, control α-6His, or no antibody. The results indicated that α-HA and α-HDAC1 antibodies were able to co-immunoprecipitate HA-INI1/hSNF5 and HDAC1 respectively (Figure 2B, lanes 3, 5, 9, and 11), while the control antibodies did not (Figure 2B, lanes 4, 6, 10, and 12). Additionally, α-HA antibodies co-immunoprecipitated BRG1, consistent with INI1 being part of the SWI/SNF complex (Figure 2B, lanes 3 and 9). We probed the above co-immunoprecipitates with antibodies against CHD3, an exclusive component of NuRD complex. While α-HDAC1 antibody co-immunoprecipitated CHD3, α-HA antibody did not (Figure 2B, lanes 5 and 11). Furthermore, another component of NuRD complex, MTA1 was also not pulled down by HA-INI1 in the same immunoprecipitates (data not shown). These results suggested that INI1/hSNF5 specifically associates with the Sin3a-HDAC1 complex.

To further establish that components of Sin3a-HDAC1 complex associate with INI1, we carried out co-immunoprecipitation experiments in the presence and absence of HIV-1 vectors, and a plasmid expressing FLAG-INI1. Co-immunoprecipitation experiments with α-FLAG antibodies indicated an association of SAP18 and HDAC1 with INI1 (Figure 2C, lane 5). Interestingly, association of these components and Sin3A was enriched when cells were cotransfected with HIV-1 vector (Figure 2C, compare lanes 5 and 7). This enrichment was not due to an increase in the level of these components in the presence of HIV-1 vector, as the input control from cells expressing Flag-INI1 with or without co-transfection of HIV-1 vectors indicated identical levels of expression of INI1, BRG1, SAP18, HDAC1 and Sin3A (Figure 2C, compare lanes 1 and 2, in the presence and absence of HIV-1). Furthermore, immunoprecipitation with α-FLAG antibodies resulted in similar amounts of FLAG-INI1 both in the presence and absence of viral vectors (Figure 2C, compare lanes 5 and 7). These results suggested that association of SAP18, HDAC1 and Sin3a is preferentially increased in the presence of HIV-1, despite the presence of similar levels of these proteins (Figure 2C, compare lanes 5 to 7). This is consistent with the idea that HIV-1 IN also binds to SAP18. Since both IN and INI1 bind to SAP18, it is possible that association of INI1 with SAP18-Sin3a-HDAC1 complex is increased upon HIV-1 infection. In fact, we have found that co-expression of IN with INI1 increases the ability of INI1 to co-immunoprecipitate HDAC1, consistent with our hypothesis (Figure S2). Furthermore, we found that co-expression of GFP-IN with FL-SAP18 in INI1−/− MON cells results in weak association of IN with SAP18 in the absence of INI1 (Figure S3). As a control, we demonstrate that BRG1, a known protein associated with INI1, co-immunoprecipitates equally well in the presence and absence of HIV-1 (Figure 2C, compare lanes 5 and 7).

There are at least four classes of histone deacetylase (HDAC) enzymes, grouped according to their homology to yeast histone deacetylases. Class I HDACs (HDAC1, 2, 3 and 8) are homologous to yeast Rpd3 and are localized in the nucleus, with the exception of HDAC3, which localizes to both nucleus and the cytoplasm [20]. Among these, HDAC1 and HDAC2 are part of the Sin3A-HDAC1 complex. To further test the specificity of association of various HDACs with INI1, we probed the INI1 co-immunoprecipitates with HDAC3, a class I HDAC, not present in the Sin3a-HDAC1 complex. Our results indicated that HDAC3 was not co-immunoprecipitated by FLAG-INI1 in the presence or absence of HIV-1 (Figure 2C, lanes 5 and 7). Furthermore, the specificity of α-FLAG antibodies to pull down FLAG-INI1 was indicated by the fact that no co-immunoprecipitation of components of Sin3a-HDAC1 complex was obtained by α-FLAG antibodies in cells expressing HA-INI1 (Figure 2C, lane 9). These results together indicate a selective association of INI1 with the components of SIN3a-HDAC1, and enhancement of this association in the presence of HIV-1 proteins.

To further confirm that IN, INI1 and SAP18 form a complex *in vivo*, we carried out co-immunoprecipitation studies using cells transiently transfected with combinations of either YFP-IN and HA-INI1, HA-INI1 and FLAG-SAP18, YFP-IN and FLAG-SAP18 or all the three proteins simultaneously (Figure 2D–G). The antibodies to FLAG, HA, or GFP (as they are cross reactive with YFP) were used for the co-immunoprecipitation experiment. We found that α-FLAG antibodies could co-immunoprecipitate FLAG-SAP18 and HA-INI1 (Figure 2D), α-HA antibodies could co-immunoprecipitate HA-INI1 and YFP-IN (Figure 2E), and α-GFP antibodies could co-immunoprecipitate YFP-IN and FLAG-SAP18 (Figure 2F), confirming the interaction of pairs of these proteins, *in vivo*. Finally, we found that α-HA antibodies could simultaneously co-immunoprecipitate HA-INI1, FLAG-SAP18 and YFP-IN consistent with the idea that these proteins form a ternary complex in vivo (Figure 2G). To further establish the stringency of our immunoprecipitation reactions, we subjected the α-GFP immunoprecipitates from the cells transfected with YFP-IN to silver staining. We found that only a select set of protein bands specific to α-GFP lane was observed, indicating the stringency of immunoprecipitation reactions (Figure S4).

### Components of the Sin3A-HDAC1 complex are specifically incorporated into HIV-1 but not SIV_mac_ particles in an IN-dependent manner

**Figure 3:**
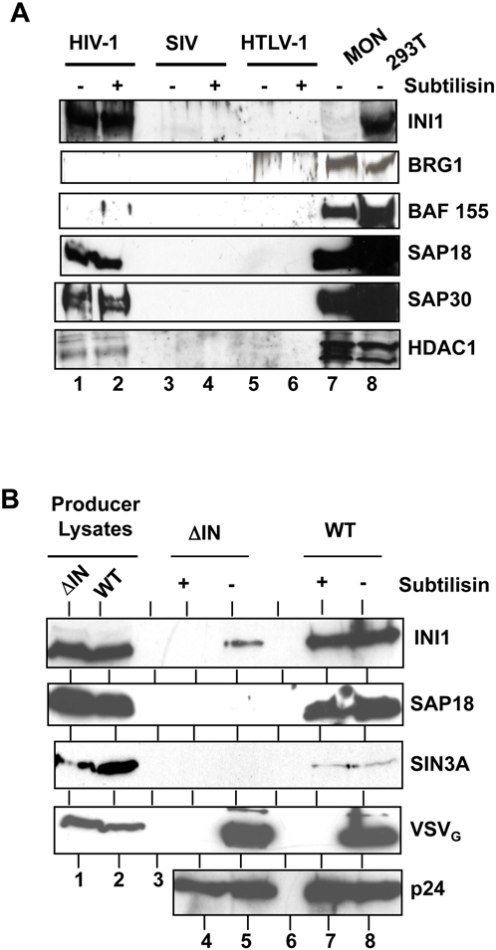
Specific IN-dependent incorporation of the INI1-associated components of SIN3A/HDAC1 complex into HIV-1 virions. (A) Incorporation of components of SIN3A/HDAC1 complex into primate retroviral particles such as HIV-1_MN_, SIV_mac_, and HTLV-1. Immunoblot of purified subtilisin-treated virions (∼125 µg each/lane), sequentially probed using α-INI1, α-BRG1, α-BAF155, α-SAP18, α-SAP30, and α-HDAC1 antibodies. (B) IN protein is required for incorporation of INI1/hSNF5 and associated components into HIV-1 virions. Purified and subtilisin-treated, wild type, and ΔIN (lacking IN) virion particles were sequentially immunoblotted using α-INI1, α-SAP18, α-SIN3A, α-VSV_G_, and α-p24 antibodies. Note that INI1, SAP18, and SIN3a are absent in virion treated with subtilisin (lane 4).

Since INI1 is specifically incorporated into HIV-1 virions and the other components of SWI/SNF complex are not, we examined the possibility that INI1-associated components of SIN3a-HDAC1 complex could be recruited to HIV-1 virions. HIV-1 virions were purified, treated with subtilisin and subjected to sequential immunoblot analysis using antibodies to components of SWI/SNF complex and that of Sin3a-HDAC1 complex. The results of these analyses indicated that while INI1/hSNF5, SAP18, SAP30, and HDAC1 were incorporated into virions, the components of the SWI/SNF complex were not (Figure 3A, lanes 1 and 2). Previously, we had reported that INI1 is not incorporated into other lentiviral or retroviral particles including SIV-1, HTLV-I, and MuLV [7]. Therefore, we examined purified and subtilisin treated preparations of SIV and HTLV-I virions for the presence of components of Sin3a-HDAC1 complex. We found that while INI1/hSNF5, SAP18, SAP30, and HDAC1 were clearly present in HIV-1 virions, they were absent from SIV_mac_ and HTLV-1 virions (Figure 3A, lanes 3–6). Furthermore, BRG1 and BAF155, the two core components of the SWI/SNF complex were absent from all virion preparations (Figure 3A, lanes 1–6). These results indicate that INI1 and its associated components of Sin3a-HDAC1 complex are specifically incorporated into HIV-1 virions.

To determine if the IN-INI1 interaction mediates specific recruitment of the Sin3a-HDAC1 complex, we generated virions lacking IN protein. Immunoblot analysis of virions lacking IN indicated that while wild type virions contained significant levels of INI1, SAP18, and SIN3a, none of these proteins were present in the subtilisin-treated virus particles lacking IN (Figure 3B, compare lanes 4 with 7). Thus, INI1 and its associated Sin3a-HDAC1 complex are specifically recruited into HIV-1 virions in a manner dependent on IN. This is consistent with the observation that IN binds to both INI1 and SAP18 *in vitro* (Figure 3B).

### HDAC1 activity is associated with HIV-1 virions

Recent findings have established a complex interplay between host cellular proteins including HDACs, histone acetyl transferase (HAT), SWI/SNF complex and HIV-1 Tat during HIV-1 LTR transcription [21]–[23]. It has been established that nucleosome organization at the LTR and proviral transcription is modulated by acetylation and deacetylation of histones [24]. While these histone modifications, occurring at the LTR promoter site, have been well established, our results indicate an association of the Sin3a-HDAC1 complex within purified HIV-1 virions. To our knowledge, specific recruitment of HDAC proteins to virions has not been documented before, either for HIV-1 or any other virus. Therefore, we decided to further investigate specifically the role of virion-associated HDAC1 in HIV-1 replication.

**Figure 4:**
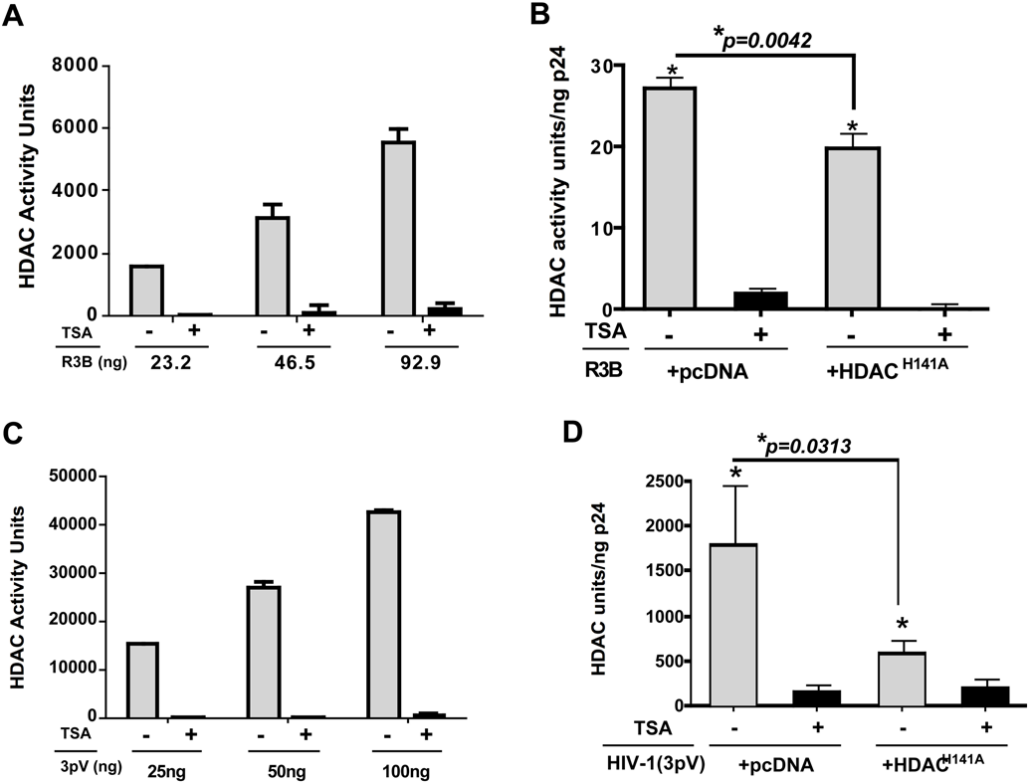
HDAC activity is associated with HIV-1 virions. Graphic representation of TSA-sensitive HDAC activity associated with HIV-1 virions, determined using a fluorimetric analysis. (A,B) HDAC activity in HIV-1_R3B_ (A) and three plasmid-based HIV-1 [(3pV, (B)] viral preparations. (C,D) Effect of incorporation of a catalytically inactive dominant negative mutant, HDAC1^H141A^ on HDAC activity associated with HIV-1 3pV (C) and HIV-1_R3B_ (D) virions. The bar graphs represent mean of three to four independent experiments +/−SEM.

We first examined for the presence of deacetylase activity in HIV-1 virion preparations to ensure that the Sin3a-HDAC1 complex is catalytically active. A fluorescence-based assay was used to detect the presence of deacetylase activity in two different preparations of virions including HIV-1_R3B_, and three-plasmid based HIV-1 vectors, using histones as substrates (Figure 4A and B). The results indicated the presence of HDAC activity that was above background and directly proportional to the amount of virus assayed. This activity was sensitive to 4 µM TSA (trichostatin A), a non-specific inhibitor of deacetylase activity (Figure 4A and B). The HDAC activity present in mock varied from batch to batch (perhaps due to the presence of cellular HDACs). Therefore, the HDAC activity of each virus preparation was normalized by subtracting mock activity. Furthermore, to eliminate the contaminating cellular HDAC proteins, we treated both virion and mock preparations with subtilisin and found that virion preparations have subtilisin-resistant HDAC1 activity, while mock preparations do not, indicating that HDAC1 activity is incorporated specifically within the virion cores (Figure S5). It should be noted that deacetylase activities of different virion preparations varied considerably depending on the type of virus, the HDAC1 activity kit and sample preparation. The three plasmid-based vectors demonstrated highest activity (200–3000 units/ng p24) as compared to R3B (20–400 units/ng p24). We also attempted to determine the HDAC1 activity associated with HIV-1 virions produced in INI1−/− MON cells, however, these results were inconclusive due to low virus yield and consequent high background (data not shown).

The fluorescence based deacetylase activity assay is relatively non-specific and will not distinguish between different HDACs. Based on our co-immunoprecipitation studies, INI1 appears to specifically associate with the Sin3a-HDAC1 complex. To investigate if deacetylase activity associated with HIV-1 virions is due to the recruitment of HDAC1, we employed an HDAC1 mutant containing a single amino acid substitution in the catalytic site (HDAC1^H141A^). This mutation renders HDAC1 protein catalytically inactive without impairing its structure, stability or association with other components of the Sin3a-HDAC1 complex [25]. Three-plasmid based HIV-1 virions were produced either in the presence of empty vector or in the presence of a vector expressing Flag-HDAC1^H141A^. The virions produced were then normalized for p24 and were assessed for the presence of deacetylase activity using a fluorescence-based assay, as above. The results of these analyses indicated that while virions produced in the presence of empty vectors harboured significant HDAC activity above the background, the virions (normalized for p24) produced in the presence of HDAC1^H141A^ demonstrated 3–10 fold statistically significant decrease (*p = 0.0313*) in HDAC activity compared to the controls (Figure 4C and Table S1). These results established that the deacetylase activity associated with HIV-1 virions is mainly contributed by HDAC1 protein and that the active HDAC1 and the associated proteins are selectively recruited into HIV-1 virions. We further assessed the activity of full-length molecular clones HIV-1_R3B_ produced in the presence or absence of HDAC1^H141A^ after normalizing the virions for p24. We found that there is a statistically significant decrease (*p = 0.004*), albeit to a lesser degree, in activity associated with HIV-1_R3B_ produced in the presence of HDAC1^H141A^ (Figure 4D).

### Requirement of HDAC1 for early events of HIV-1 replication

**Figure 5:**
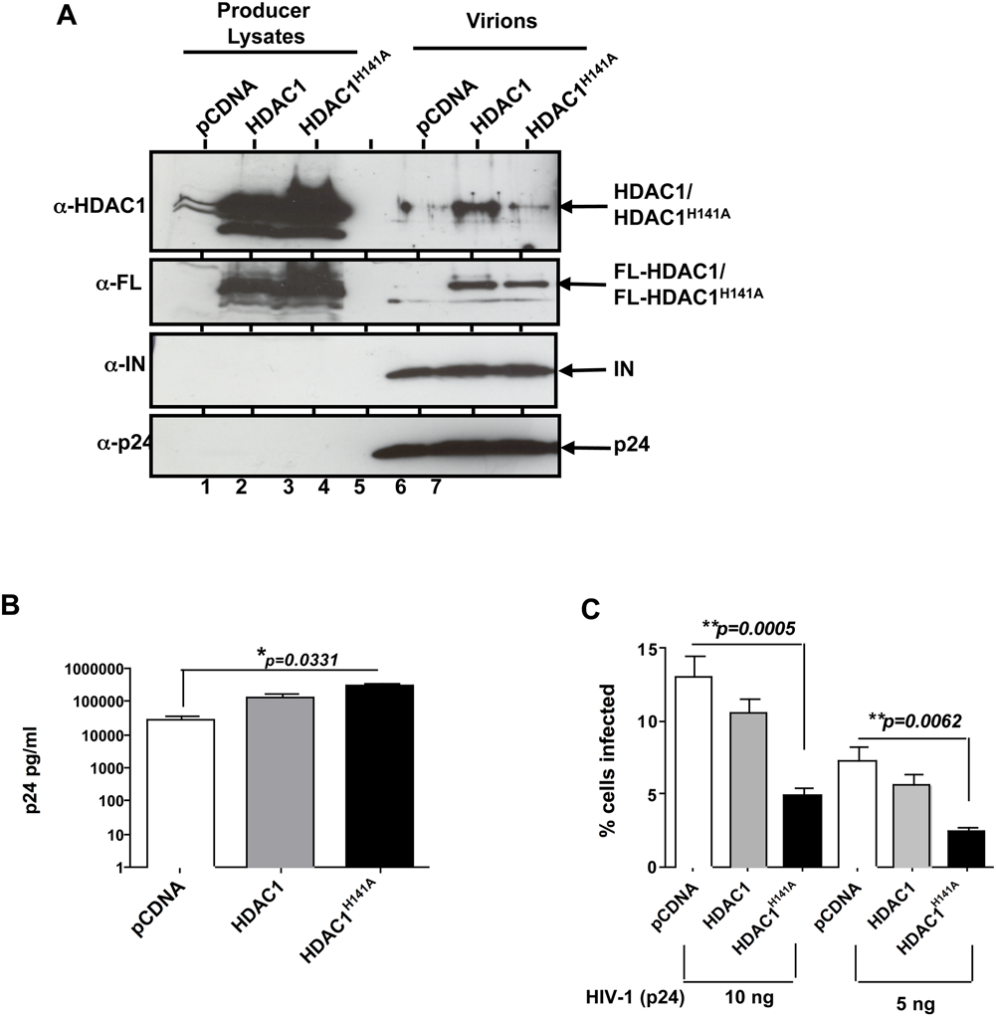
Virus-associated HDAC1 activity is required for HIV-1 replication in target cells. (A) Immunoblot analysis of virions produced in the presence of either an empty vector or vectors expressing FLAG-HDAC1 or FLAG-HDAC1^H141A^, using indicated antibodies. (B) Effect of expression of FLAG-HDAC1 or FLAG-HDAC1^H141A^ vectors on the production of HIV-1 virus particles. The bars represent average p24 values obtained from three independent experiments (+/−SEM). (C) Effect of incorporation of HDAC1^H141A^ on the infectivity of HIV-1 virions. Graphic representation of the % GFP positive (infected) cells obtained when 293T cells were exposed to three plasmid based HIV-1 harbouring either FLAG-HDAC1 or FLAG-HDAC1^H141A^ (average of 3 independent experiments +/−SEM).

To determine the functional significance of virion-associated HDAC1 for HIV-1 replication, we tested the effect of expressing a catalytically inactive mutant, HDAC1^H141A^ that acts as a dominant negative mutant, on HIV-1 p24 release and infectivity. Pharmacological agents such as TSA are not specific to HDAC1 and would inhibit all Class I and Class II HDACs. The effect of HDAC1^H141A^ is specific and it impairs neither the structure nor the stability of the protein, nor the association of the mutant protein with other components of the Sin3a-HDAC1 complex [25]. Therefore, by expressing this mutant in the producer cells, we could incorporate it into the virions and selectively test the requirement of virion-associated HDAC1. We produced HIV-1 virions in the presence of HDAC1^H141A^ or the wild type HDAC1 as control, by co-expressing these proteins in the producer cells along with the viral vectors. Immunoblot analysis of normalized amounts of purified virions produced from these cells revealed specific incorporation of equivalent amounts of FLAG-tagged HDAC1 or HDAC1^H141A^ proteins in the virions (Figure 5A, lanes 6 and 7). Furthermore, re-probing the same blot with antibodies specific to IN and p24 indicated identical amounts of these proteins, suggesting that incorporation of HDAC1 mutant does not impair the level of these viral proteins (Figure 5A).

To assess the effect of HDAC1^H141A^ expression on p24 release, virions were collected from cells expressing empty vector, HDAC1 or HDAC1^H141A^ and subjected to p24 analysis. We observed a significant increase in viral p24 release when FLAG-HDAC1^H141A^ was expressed in the producer cells (∼5–10 fold compared to the empty vector control, *p = 0.0331*, Figure 5B and Table 1). There was a slight increase in p24 release when wild type HDAC1 was expressed as compared to empty vector, but the difference was much less (*p = 0.0948*). We next assessed the infectivity of virions produced in the presence of either FLAG-HDAC1 or FLAG-HDAC1^H141A^ for their ability to infect target cells, after normalizing for p24 levels. We found that virions produced in the presence of FLAG-HDAC1^H141A^ were significantly reduced in their infectivity (over 3–10 fold reduction; *p = 0.0005 and 0.0062* for 10 ng and 5 ng p24 of virions, respectively) compared to virions produced in the presence of empty vector, given the identical amount of p24 (Figure 5C and Table 2). There was a slight decrease in the infectivity of virions produced in the presence of wild type HDAC1, but the difference was much less compared to virions produced in the presence of FLAG-HDAC1^H141A^ (Figure 5C). Since these virions were produced in cells containing endogenous wild type HDAC1, it is likely that we were unable to completely eliminate encapsidation of this protein into virions. This is reflected by the presence of significant residual HDAC1 activity within virions harbouring mutant FLAG-HDAC1^H141A^ (Figure 4D). Overall, these results indicated that virion-associated HDAC1 activity is required for efficient infectivity of HIV-1.

**Table 1:** Effect of co-transfection of HDAC1 and HDAC1^H141A^ on p24 release.

Viral DNA (10 µg)	Plasmid co-transfected	Mean p24 ng/ml*	+/−SEM**
3pV	pCDNA	29509	9299
3pV	pHDAC1	134460	8802
3pV	pHDAC1**^H141A^**	279801	63706

*: Indicates mean of six different experiments done in triplicates. The average of each experiment was computed to obtain mean value.

**: SEM = standard mean of error.

**Table 2:** Effect of co-transfection of HDAC1 and HDAC1^H141A^ in the producer cells on viral infectivity.

Viral preparation	Amount of virus used (p24)	% cells infected (mean)*	+/−SEM**
3pV+pCDNA	10 ng	13.07	1.413
3pV+pHDAC1	10 ng	10.51	1.116
3pV+pHDAC1**^H141A^**	10 ng	4.871	0.6632
3pV+pCDNA	5 ng	7.216	1.116
3pV+pHDAC1	5 ng	5.569	0.8977
3pV+pHDAC1**^H141A^**	5 ng	2.414	0.3753

*: Indicates mean of six different experiments done in triplicates. The average from each experiment was computed to obtain mean value.

**: SEM = standard mean of error.

### HDAC1 activity does not modulate SIV replication

**Figure 6:**
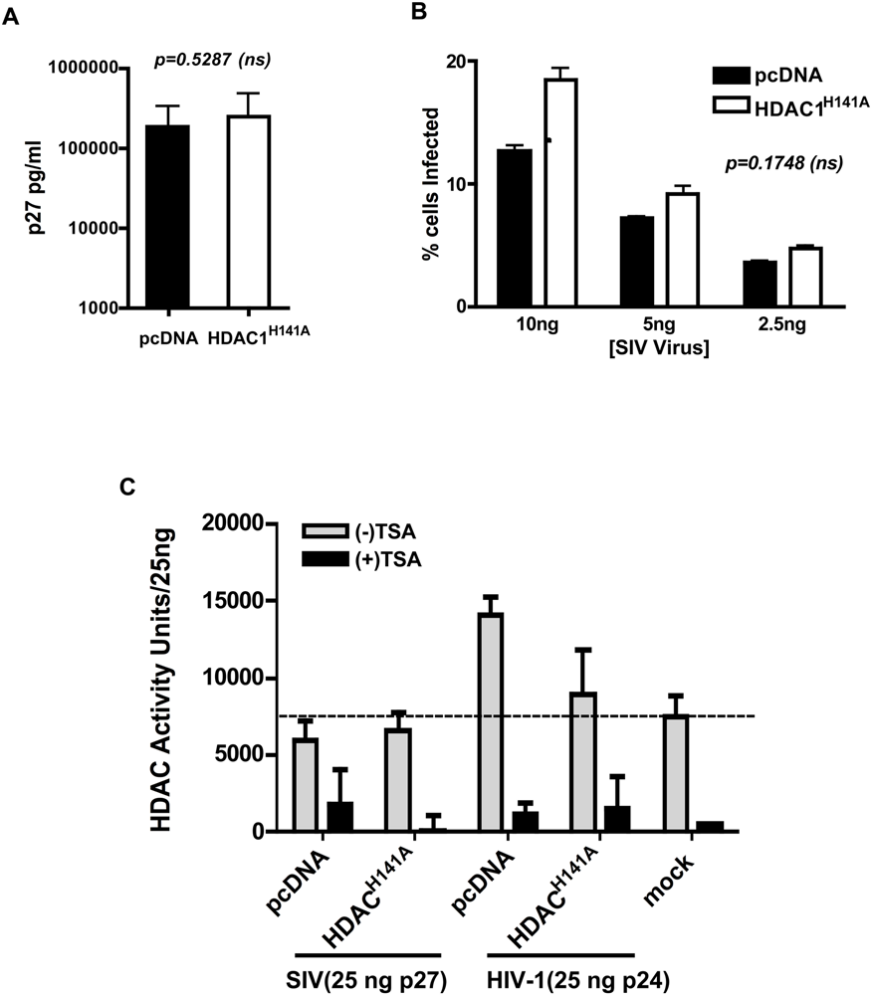
Effect of HDAC1^H141A^ on SIV replication. (A) Effect of expression of FLAG-HDAC1^H141A^ vectors on the production of SIV-1 particles. The bars represent average p27 values obtained from three independent experiments (+/−SEM). (B) Effect of incorporation of HDAC1^H141A^ on the infectivity of SIV virions. Graphic representation of the % GFP positive (infected) cells obtained upon infection of 293T cells with SIV vector produced in the presence or absence of pFLAG-HDAC1^H141A^ (average of 3 independent experiments +/−SEM). (C) Comparative analysis of HDAC1 activity associated with HIV-1 and SIV virions in the presence and absence of HDAC1^H141A^. Equal amounts of SIV (in p27 ng) and HIV-1 (in p24 ng) were subjected to HDAC activity assay along with the mock-transfected culture supernatants. Note: the HDAC activity detected in SIV is similar to that of mock control, and HIV-1 harbours higher levels of HDAC activity compared to that of SIV and mock.

INI1 interacts with HIV-1 but not SIV IN [7]. Furthermore, INI1 and the components of Sin3A-HDAC1 complex are incorporated into HIV-1 but not SIV_mac_ virions (Figure 3A). To further confirm the functional specificity of the Sin3A-HDAC1 complex to HIV-1, we tested the effect of FLAG-HDAC1^H141A^ on the production and infectivity of a VSV_G_ pseudotyped SIV-based vector, carrying a GFP reporter gene [26]. SIV virions were produced in the presence and absence of FLAG-HDAC1^H141A^ in 293T cells. We observed that expression of FLAG-HDAC1^H141A^ did not significantly increase SIV particle production as compared to empty vector control (*p* = 0.5287, Figure 6A). Contrary to the effect on HIV-1, presence of FLAG-HDAC1^H141A^ did not significantly affect the infectivity of SIV virions (*p* = 0.1748, Figure 6B). To further confirm that HDAC1 is not incorporated into SIV, we carried out a fluorescent-based deacetylase activity assay as described above using SIV and HIV-1, produced in the presence or absence of FLAG-HDAC1^H141A^. We found that SIV virions produced in the presence or absence of FLAG-HDAC1^H141A^ exhibited deacetylase activity similar to that of the mock control (*p* = 0.5916, Figure 6C). Unlike SIV, HIV-1 virions exhibited higher deacetylase activity compared to mock control (Figure 6C). Furthermore, presence of FLAG-HDAC1^H141A^ decreased the HDAC1 activity in HIV-1 virions (*p* = 0.0265, Figure 6C). These results establish that HDAC1 activity is specifically associated with HIV-1 but not SIV, consistent with the selective incorporation of the Sin3A-HDAC1 complex into HIV-1.

### HIV-1 virion-associated HDAC1 activity is required for a post-entry step during or before early reverse transcription

**Figure 7:**
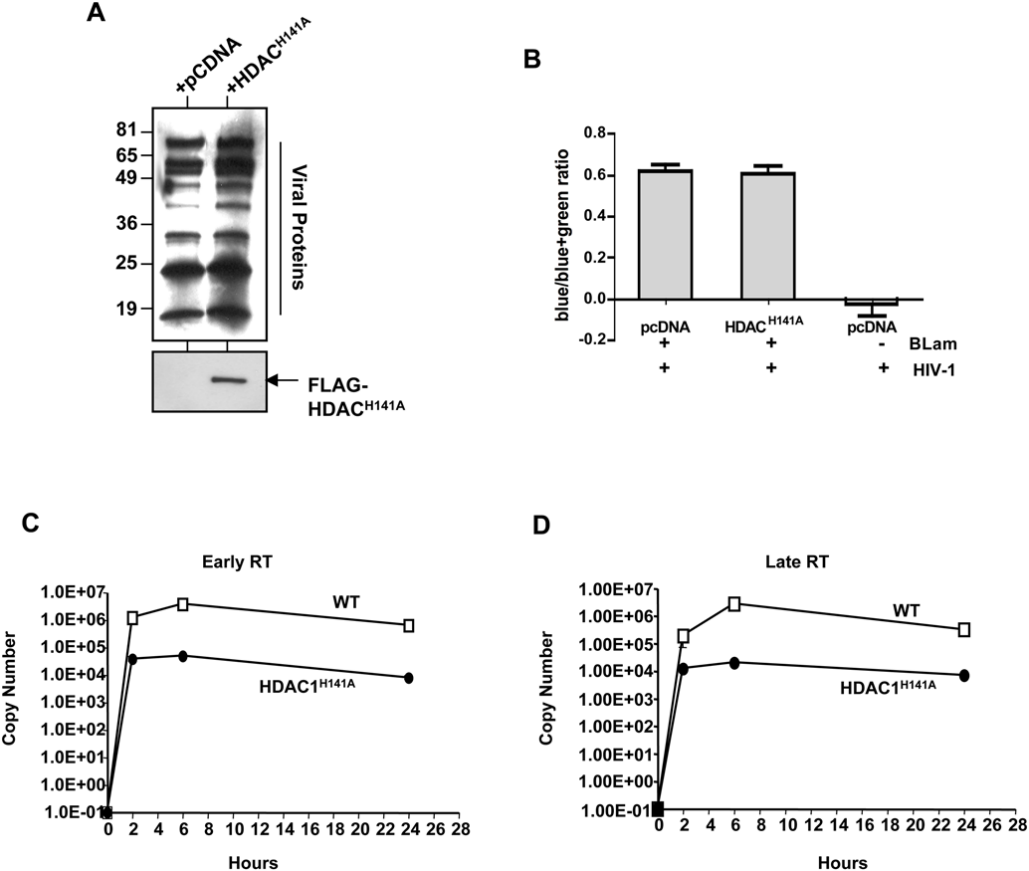
Functionally inactive HDAC1^H141A^ inhibits early post-entry events of HIV-1 replication. (A) Immunoblot analysis of virion-associated proteins produced in the presence of FLAG-HDAC1^H141A^. Equal amounts of purified and concentrated virions were loaded and subjected to immunoblot analysis using IgG from HIV-1 patient serum and α-FLAG antibodies. (B) Effect of incorporation of HDAC1^H141A^ on the entry and fusion of HIV-1. Virions were produced incorporating Vpr-BLAM, and equal p24 amount of virions were used to infect SupT1 cells to determine the ratio of blue cells to green+blue cells. (C,D) Graphic representation of copy number of early (C) and late (D) reverse transcription products formed upon infection of 293T cells with equal amounts of virus harbouring FLAG-HDAC1^H141A^. The average copy numbers of RT products (Y-axis) were determined by quantitative real time PCR at various time points post-infection (X-axis). Data represents average of three independent experiments (*+/−*SD).

The results of the above experiments strongly suggest that virion-associated HDAC1 is required for early events of HIV-1 replication, which include virus entry, uncoating, reverse transcription, nuclear translocation and integration. To rule out the possibility that incorporation of HDAC1 mutant may affect general processing or incorporation of viral proteins, the purified viral preparations were subjected to immunoblot analysis using anti-HIV-1 serum. The results indicated that the general processing of viral proteins was not affected in the presence of FLAG-HDAC1^H141A^ (Figure 7A). To determine if the incorporation of FLAG-HDAC1^H141A^ blocks virus entry, we carried out a FRET-based entry assay, using Vpr-fused β-lactamase (BLAM) [27]. In this assay, entry of HIV-1 leads to cleavage of a substrate by virion-associated β-lactamase, which can be quantitated by observing changes in FRET. Cells successfully infected with HIV-1 emit a blue FRET whereas cells without viral entry remain green. We produced HIV-1 virions in the presence and absence of FLAG-HDAC1^H141A^, simultaneously incorporating Vpr-BLAM. Normalized amounts of virions containing Vpr-BLAM were then used to carry out the entry assay. We quantitated blue cells and determined the ratio of blue cells (successful HIV-1 entry) to blue plus green cells (total number of cells). We found that virions containing FLAG-HDAC1^H141A^ exhibited the same level of entry compared to that of the empty vector control, indicating that FLAG-HDAC1^H141A^ does not affect HIV-1 entry (Figure 7B).

We next examined if replication of virions harbouring FLAG-HDAC1^H141A^ is blocked at a post entry step such as uncoating, early or late reverse transcription. A quantitative-real-time PCR (Q-PCR) analysis was carried out using DNA isolated from cells infected with equal amounts of virions normalized by p24 ELISA. HIV-1 infection resulted in synthesis of early RT products within 2 h, with a peak at 6 h post-entry (Figure 7C). We found that HIV-1 virions harbouring FLAG-HDAC1^H141A^ exhibited a ∼10 fold decrease in the amount of early RT products at all time points tested compared that of control virions (Figure 7C). A similar trend was observed for late RT products using qPCR. Virions harbouring FLAG-HDAC1^H141A^ exhibited ∼10 fold decrease in late RT products compared to that of the controls (Figure 7D). The levels of reduction in early and late reverse transcription products are in agreement with the reduction in the level of infectivity of virions harbouring HDAC1^H141A^. These results strongly suggest that the defect in infectivity of virions harbouring HDAC1^H141A^ is due to a block at a step after entry and before or at early reverse transcription.

### RNA interference analysis to determine the requirement of HDAC1 to HIV-1 infection

**Figure 8:**
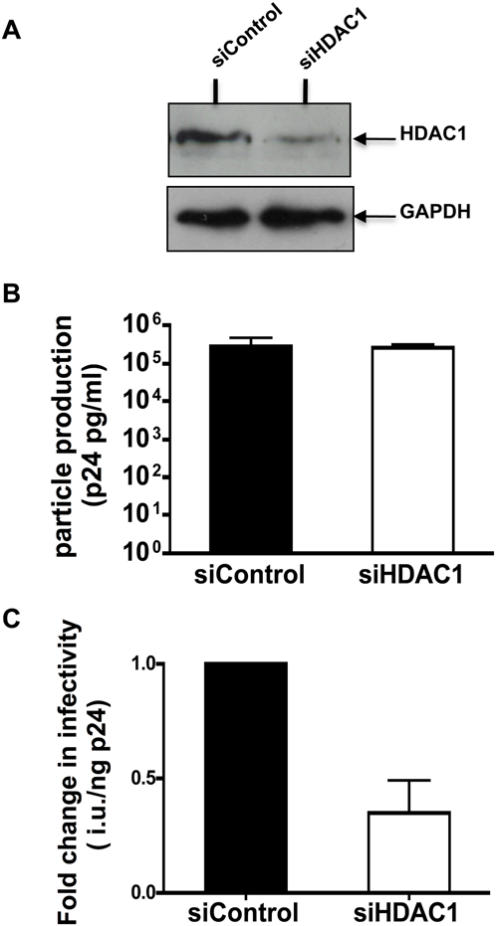
siRNA knockdown of HDAC1 in virus producer cells results in decreased infectivity of HIV-1 in target cells. Analysis of the effect of HDAC1 knockdown on viral particle production and subsequent infectivity of HIV-1: (A Immunoblot analysis of 293T cells exposed to either siHDAC1 or siControl. (B) Effect of HDAC1 knockdown on viral particle production. Viral supernatants from cells co-transfected with either siHDAC1 or siControl along with HIV-1_R3B_ were subjected to p24 analysis. Bars represent average p24 ng/ml values obtained from three independent experiments. (C) Analysis of infectivity of normalized amounts of HIV-1_R3B_ virus produced in cells transfected with either siControl or siHDAC1 using GHOST reporter cells. Bars represent the average fold change in infectious units/ng p24 from three independent experiments.

To further substantiate a link between virion-associated HDAC1 activity and infectivity, we carried out an RNA interference analysis. 293T cells were first transfected with either control siRNA or siRNA against HDAC1. These cells were then co-transfected 24 hours later with siRNA a second time, along with HIV-1_R3B_ DNA. To determine if knock-down of HDAC1 affects viral infectivity, viral supernatants were collected and subjected to p24 ELISA. Equal amounts of p24 isolated from control and HDAC1 siRNA transfected cells were subjected to HDAC activity and infectivity assays. Western analysis indicated a partial knock-down of HDAC1 from the producer cells (Figure 8A). Knock-down of HDAC1 did not affect particle production (Figure 8B). However, virions produced in the knock-down cells exhibited a decrease in infectivity compared to that of the controls (Figures 8C). Analysis of virions produced from the knock-down cells indicated a decrease in HDAC1 activity that varied from experiment to experiment (representative experiments are provided in Figure S6A). Interestingly, the fold decrease in HDAC1 activity correlated to fold decrease in infectivity (Figure S6A and Figure S6B). These results strongly indicate that virion-associated HDAC1 activity plays an important role in determining the degree of virion infectivity.

## Discussion

Our results provide a novel paradigm for the role of INI1 and components of the Sin3a-HDAC1 complex in HIV-1 replication. Interaction of the SWI/SNF complex with components of the HDAC1 complex has been previously reported [14],[28]. Our results demonstrate that INI1/hSNF5 and IN directly interacts with SAP18, a component of Sin3a-HDAC1 complex. Furthermore, for the first time, we find that components of the Sin3a-HDAC1 complex are selectively recruited into HIV-1 virions. It is also interesting to note that core components of the SWI/SNF complex such as BRG1, BRM, BAF155 and BAF170 are absent from HIV-1. To our knowledge, this is the first report where association of INI1 with components of an HDAC1 complex has been demonstrated in the absence of the SWI/SNF complex. We believe that further analysis of this association will lead to uncovering novel biological functions of INI1 and the Sin3a-HDAC1 complex.

The recruitment of the Sin3a-HDAC1 complex appears to be specific to HIV-1 and dependent on IN. However, more experiments are needed to determine if IN alone can recruit the complex into virions and if INI1 is required for this function. Furthermore, we have demonstrated that HIV-1 virions harbour deacetylase activity, which is reduced when a catalytically inactive mutant, HDAC1^H141A^, is incorporated into virions. To our knowledge, this is the first report of an HDAC1 activity associated with HIV-1 particles. Expression of HDAC1^H141A^ in producer cells leads to two distinct effects: (i) enhancement of p24 release; and (ii) a statistically significant decrease in the infectivity of the particles produced. These effects appear to be specific to HIV-1 and not SIV. Furthermore, knocking down HDAC1 in the producer cells also resulted in decrease of virion-associated HDAC1 activity and reduction in infectivity of those particles, consistent with the results of the HDAC1^H141A^ mutant.

We surmise that the increase in particle production observed when HDAC1^H141A^ is expressed is due to the derepression of viral and/or cellular transcription. We found that intracellular p24 was indeed increased when HDAC1^H141A^ mutant was expressed (data not shown), consistent with this idea. The decrease in infectivity of HIV-1 virions harbouring HDAC1^H141A^ is directly correlated to a decrease in virion-associated deacetylase activity. While the HIV-1-associated deacetylase activity is not required for entry into target cells, it appears to be required for early reverse transcription. These results suggest that virion-associated Sin3a-HDAC1 complex is required for either efficient reverse transcription or uncoating after entry. It is interesting to note that virions produced in *INI1*−/− cells are defective for reverse transcription, and that INI1 is associated with the HIV-1 reverse transcription complex [29],[30]. Furthermore, many laboratories including ours, have demonstrated that IN directly interacts with RT and that mutants of IN affect RT function [31]–[34]. These observations raise the possibility that one mechanism by which IN influences RT function is via the recruitment of the Sin3a-HDAC1 complex through its direct interaction with SAP18 and INI1. Future experiments to address the relationship between binding of IN to INI1 and SAP18, and its influence on RT function, are likely to shed new light on the dynamic and functional interaction between HIV-1 RT, IN and IN-associated complexes.

Our results also parallel an earlier report where, it was demonstrated that cellular Sin3a protein was required for retrotransposition of *Schistosaccharomyces pombe* transposon, Tf1. In the Tf1 system, Sin3a appears to be required for a post reverse transcription event and mutations in *Sin3a* block a step before nuclear entry [35]. This genetic study indicated that the Sin3a-HDAC1 complex is required for a step other than viral transcription in retroviral/retrotransposon replication. However, in the case of HIV-1, we have found that the Sin3A-HDAC1 complex is required for early reverse transcription. Future experiments are required to uncover the exact mechanism by which virion-associated HDAC1 modulates post entry events of HIV-1.

INI1 appears to play a complex role in HIV-1 replication due to its multitude of effects. Studies using INI1 dominant negative mutants as well as p24 release in *INI1*(−/−) cells indicated that INI1 is required for HIV-1 replication [6],[29]. Furthermore, particle produced in INI1−/− MON cells were defective for infection and were blocked at the stage of reverse transcription [29]. These latter results are consistent with the current report in that virion-associated HDAC1 activity is required for efficient early events of HIV-1. On the contrary, Maroun et al reported that while knocking-down INI1 in the producer cells has no effect, knocking-down INI1 in the target cells increases infection of the virus, indicating that it is an anti-viral protein [12]. In this report, virions produced in the INI1 knock-down cells were not analyzed for the presence of INI1 and hence the data is not interpretable. Furthermore, it is possible that INI1 was not completely knocked-down in the producer cells and that INI1 in the target cells plays different roles to INI1 in the producer cells.

Studies on siRNA-mediated knock-down of INI1 is complicated due to the fact that INI1 is a tumour suppressor and it is required for the survival of many cell types. Complete knock down of *INI1* results in flat cell formation and apoptosis in HeLa cells [36]. Furthermore, other studies demonstrated that: (i) homozygous deletions of *INI1* causes embryonic lethality at the peri-implantation stage; (ii) embryonic cells lacking *INI1* undergo apoptosis; and (iii) conditional knock-out of *INI1* results in massive apoptosis [37]–[41]. These studies highlight the fact that knocking-down INI1 may cause cell lethality. Inefficient transient knockdown of *INI1* can be achieved in cells, but may not be suitable for assessment of the effect of its loss, as it has been documented for LEDGF that even a small amount of the protein is sufficient to provide the desired function during HIV-1 replication [42]. In addition to playing a role in late events during p24 release, *INI1* binds Tat and is required for Tat-mediated LTR transcription [8]–[11]. Because of these reasons, it is important to first to segregate multiple effects of INI1 on cellular and viral functions to fully comprehend its effect on HIV-1 replication.

Based on our current study, we hypothesize that while the virion-associated INI1-Sin3a-HDAC1 complex may affect early reverse transcription, cellular INI1 associated with the SWI/SNF complex may have antiviral effects in target cells. It is possible that deacetylation of a substrate (an acetylated viral or cellular protein) by virion-associated HDAC1, is required to mediate efficient early reverse transcription of HIV-1. The acetylated viral or cellular protein may block early post-entry events and deacetylation may be necessary to overcome this block. Alternatively, a viral or cellular protein required for an early event may need deacetylation to become active. While histones are classic substrates that are modified by acetylation and deacetylation, an increasing list of non-histone proteins that are similarly modified have been identified [43]. Possible candidate viral proteins include IN, although requirement of acetylation for IN activity is controversial [44],[45]. Alternatively, other viral proteins or unidentified cellular proteins could be substrates for deacetylation by the virion-associated Sin3a-HDAC1 complex.

While our studies investigate the virion encapsidation and functional significance of Sin3A-HDAC1 complex in HIV-1 replication, a recent report illustrated the association of NURD, another HDAC1 complex, with the HCMV viral protein UL38. In this case, association of UL38 with HDAC1 complex leads to inhibition of host stress response [46]. Whether association of IN, INI1 with the SAP18-HDAC1 complex also serves to inhibit host stress response remains to be determined. We have recently found that INI1 is involved in inducing an interferon response in rhabdoid tumour cells [47]. By binding to INI1 and SAP18, HIV-1 IN may inhibit anti-viral interferon response during infection. Future experiments to determine such interference of the host innate immune response (due to the binding of viral and cellular proteins such as IN, INI1, and the SAP18-HDAC1 complex), are likely to shed novel insight into complex host-virus interactions. Furthermore, we believe that strategies involving disruption of IN-INI1-SAP18-HDAC1 interactions or selective targeting of virion-associated HDAC complexes may lead to novel methods to inhibit HIV-1 replication.

## Materials and Methods

### Plasmids

pGEX-SAP18 expressing GST-SAP18 was generated by cloning into pGEX3xPL, a BamH1-EcoRI fragment obtained by PCR amplification of a full length *SAP18* EST clone (IMAGE 364760, GenBank accession number AA025356) using primers 5′-CTGACGGAAAATGAATTCAAC-3′ and 5′-GGCCGTAAGAGGATCCTGGCGGTC-3′. A plasmid encoding INI1-FLAG fusion protein, pBABEpuro-INI1-FLAG was generated as described [48]. The pBABEpuro-FLAG control plasmid was generated from pBABEpuro-INI1-FLAG by deleting INI1 fragment. The gag-pol expression vector containing IN deletion, pCMVΔR8.2ΔIN was generated as follows. First, an intermediate vector (pSP72-Cla-Sal) carrying a ClaI-SalI fragment of HIV-1 R3B virus was generated and subjected to site directed mutagenesis using the primers 5′…GCTCTCCAATTACTGTGCTAGCTCTCATGTTCTTCTTGG…3′ and 5′…CCAAGAACATGAGAGCTAGCACAGTAATTGGAGAGC…3′ to generate stop codon at the beginning of IN (pSP72-deltaIN). The *SalI-BclI* fragment containing IN mutation was isolated from pSP72-deltaIN and cloned into the pCMVΔR8.2 vector to generate pCMVΔR8.2ΔIN. The pCDNA3.1-HDAC1 and pCDNA3.1-HDAC1^H141A^ plasmids were gifts from Dr. Schreiber (Harvard Medical School). Three-plasmid HIV-1-based vectors were obtained from Dr. Trono and the SIV-based lentiviral system (pCAG-SIVgprre, pCAG4-RTR-SIV and pCL20cSLFR MSCV-GFP) was a kind gift of Dr. Nienhuis (St. Jude Children's Research Hospital). These vectors were pseudotyped by VSV_G_ (expressed from pMDG-VSV_G_). GHOST (3) X4/R5 reporter cells containing LTR-GFP (Cat# 3942) was obtained from NIH AIDS research and reference reagent program.

### Virus particle production and infection

HIV-1 virus harbouring HDAC1^H141A^ mutant and the control and wild type proteins were generated by co-transfecting the HIV-1 or SIV-based viral vectors, carrying GFP marker with FLAG-, FLAG-HDAC1 or FLAG-HDAC1^H141A^-encoding plasmids. Particle production was monitored by using p24 ELISA kit (Perkin Elmer Cat# NEK050B for HIV-1) and p27 ELISA kit (Advanced Bioscience Laboratories Inc., Kensington, MD, Cat# 5436 for SIV) and the infectivity was determined by monitoring the expression of GFP markers upon infection of target cells using FACS analysis.

### Immunoblot analysis

Concentrated and purified virions digested with subtilisin, were lysed and then subjected to immunoblot analysis using the following antibodies: monoclonal α-IN (a kind gift of Dag Helland and Alan Engelman); affinity purified polyclonal α-INI1, INI1-PB3 (Yung et al., 2001); goat polyclonal α-p24, monoclonal α-gp41, polyclonal α-p30, polyclonal α-VSV_G_ (were provided by Dr. Ott); polyclonal α-BRG1, α-BAF155, α-BAF170 (gift of Dr. Weidong Wang at National Institute on Aging, National Institute of Health). Antibodies against the following proteins and tags were purchased: SAP18 (Santa Cruz #SC-8473); HA (Santa Cruz #SC-805); SIN3A (Santa Cruz #SC-767); HDAC1 (Upstate # 06-720); HDAC3 (Upstate # 06890); FLAG (Sigma # F3165); HIV IgG (AIDS Reagent Program, catalogue # 3957); hBRM1 (BD Biosciences cat# 610389); β-Actin (Sigma #AC-15).

### Protein–protein interactions

SAP18 as an interacting partner for INI1 was isolated by using LEXADB-INI1 as a bait and screening HL60 cDNA library in yeast two hybrid system as described [18]. For GST pull down assay to analyze direct interaction between GST-SAP18 and His-INI1 and His-IN, glutathione sepharose 4B beads bound to 5 µg of either GST or GST-SAP18 were prepared as described [18] and incubated with 2 µg of either Ni-NTA purified His-IN or Ni-NTA and hydroxylapatite purified His-INI1 in buffer containing 20 mM HEPES-KOH (pH 6.8), 200 mM NaCl, 0.1 mM EDTA, 2–5 mM DTT, 0.1% IGEPAL, 100 µg/ml ethidium bromide and protease inhibitors and treated with 10 U of DNaseI. Following incubation, beads were washed 3–5× with buffer containing 20 mM HEPES-KOH (pH 6.8), 200 mM NaCl, 0.1 mM EDTA, 1 mM DTT, 100 µg/ml ethidium bromide, 0.5% IGEPAL and 1 mM PMSF. Bound proteins were resolved by SDS-PAGE and analyzed by Western blot using indicated antibodies.

### Co-immunoprecipitation analyses

#### (i) Co-immunoprecipitation of INI1 with components of HDAC1 complex

HeLa and MON cells were collected 40 hours after transfection and sonicated in 500 µl buffer G (dPBS containing 0.1% IGEPAL, 1 mM DTT, 2 mg/ml BSA, 1 mM PMSF and 1 µg/ml each of pepstatin, aprotinin and leupeptin). Lysates were pre-cleared by incubating with 30 µl of protein A sepharose (50% slurry), antibodies were added to pre-cleared lysates followed by protein A sepharose beads (30 µl of 50% slurry) and incubated overnight at 4°C. Bound proteins were washed 4 times with buffer G without BSA, separated by SDS/PAGE and subjected to immunoblot analysis. Co-immunoprecipitations of FLAG fusion proteins were carried out using FLAG Immunoprecipitation Kit (Sigma cat # FLAGIPT-1) as per manufacturer's recommendations.

#### (ii) Co-immunoprecipitation of endogenous proteins

Confluent HeLa cells were lysed in co-IP buffer containing 20 mM HEPES-KOH (pH 7.9), 150 mM NaCl, 5 mM MgCl_2_, 5 mM CaCl_2_, 0.1 mM EDTA, 1 mM DTT, 1% Triton-X, treated with 0.033 U/µl micrococcal nuclease and immunoprecipitated overnight with 1.6 µg of α-SAP18 antibody (ABCam ab31748-25) or rabbit IgG. Complexes were pulled down with Protein A Agarose and washed 3 times with wash buffer containing 20 mM HEPES-KOH (pH 7.9), 150 mM NaCl, 0.1 mM EDTA, 1 mM DTT, 1% Triton-X. Complexes were resolved by SDS-PAGE and Western blot analysis was carried out using affinity purified rabbit polyclonal α-SAP18 antibody.

#### (iii) Co-immunoprecipitation of IN-INI1-SAP18 *in vivo*

For analysis of complex formation between HA-INI1, FLAG-SAP18 and YFP-IN, 293T cells were transfected with the plasmids expressing these proteins. 48 hrs post-transfection, cells were lysed in co-IP buffer as above and treated with 0.033 U/µl micrococcal nuclease and immunoprecipitated overnight with 2 µg of indicated antibodies or control IgG. Complexes were pulled down with Protein A Agarose and washed 3–5× with wash buffer as above. Complexes were resolved by SDS-PAGE and Western blot analysis carried out using indicated antibodies. The antibodies used for immunoprecipitation were rabbit polyclonal α-HA (SantaCruz sc805), monoclonal α-FLAG (Sigma F3165), monoclonal α-GFP (Sigma G6539) and for Western blot analysis were α-HA-HRP (Sigma H6533), α-FLAG-HRP (Sigma A8592) and monoclonal α-IN antibodies.

### Quantitative real-time PCR (qPCR)

Virus stocks (5 ng p24) produced in 293T cells were treated with 50 U ml^−1^ with DNase (Roche) for 60 min at 37°C, and used to infect 2.0×10^5^ 293T cells in 6-well plates. After 2 hours of infection, the cells were washed with PBS, and incubated with fresh DMEM. Genomic DNA was isolated at various time points using DNeasy kit (Qiagen). Early HIV-1 reverse transcripts were quantified using primers ert2f, ert2r and the ERT2 probe; and late reverse transcripts were quantified using primers MH531 and MH532 respectively, as described [49] using Taqman method. Reactions were analyzed in triplicates using the ABI Prism 7700 (Applied Biosystems).

### Quantitation of HDAC activity in HIV-1/SIV virions

Virion-associated HDAC activity was measured using HDAC Fluorometric Activity Assay kit (Upstate #17-356) as per manufacturer's recommendations. The HDAC Activity assays were measured using a Victor 2 multi-well plate reader (Perkin Elmer) with an excitation and emission wavelength set to 355 and 460, respectively. The lamp energy was set to 3948 with a measurement time of 0.1 sec and an emission aperture set to normal. For determining the HDAC activity of HIV or SIV based vectors in the presence or absence of HDAC1 or HDAC1^H141A^ mutant, equal amount of virion preparations (by p24 ELISA) were subjected to HDAC1 activity assay. Mock-transfected culture supernatants were clarified in a manner similar to viral preparations and were used to determine the background level activities. HDAC activities detected in the mock samples were subtracted from the control and test samples, except in Figure 6C.

### Statistical analysis

Statistical analysis was performed using GraphPad Prism version 4.00 for Macintosh, GraphPad Software, San Diego California USA, www.graphpad.com. All data points (including outliers) were included in the analysis for significance and paired comparisons were carried out using t-statistic (two tailed) where equal variance in data between categories was assumed.

Gene symbols and nomenclature used in the manuscript have been provided in the Table S2.

## Supporting Information

Figure S1 Immunoblot analysis of virions. Purified, concentrated, and subtilisin-treated HIV-1 mn virions were immunoblotted with various antibodies. The same blot was sequentially probed with α-INI1 (A), α-BRG1 (B), α-BAF170 (C), α-BAF155 (D), α-gp41 (E), and α-gp24 (F) antibodies, as indicated. Total protein lysates from MON (INI1−/− rhabdoid cells) and 293T (INI1+/+) were used as controls.
(0.24 MB PDF)

Figure S2 IN enhances the complex formation of INI1 with HDAC1. 293T cells were transfected with FLAG-INI1 and either pCDNA or YFP-IN plasmids and subjected to immunoprecipitation with either IgG or anti-FLAG monoclonal antibody agarose, as indicated. Immunoprecipitated complexes were analyzed by anti-FLAG monoclonal, anti-GFP polyclonal, and anti-HDAC1 polyclonal antibodies. Input lysates showing equal loading for FLAG-INI1, YFP-IN, and HDAC1 are shown.
(0.14 MB PDF)

Figure S3 Interaction of IN with Sap18 in (INI1−/−) MON cells. MON cells were transfected with YFP-IN and FLAG-SAP18 plasmids and subjected to immunoprecipitation using either mouse IgG or anti-FLAG monoclonal antibody agarose. Immunoprecipitated complexes were analyzed using western blots with both anti-GFP polyclonal and anti-FLAG monoclonal antibodies. Input lysates showing equal loading for both YFP-IN and FLAG-SAP18 are shown.
(0.15 MB PDF)

Figure S4 Silver stain analysis of immunoprecipitations to determine the specificity of interaction. Silver stain analysis of immunoprecipitations to determine the specificity of interaction. 293T cells were transfected with YFP-IN plasmid and subjected to immunoprecipitation using either mouse IgG or anti-GFP monoclonal antibody. Immunoprecipitated complexes were analyzed by SDS-PAGE followed by silver staining. Position of YFP-IN, IgG heavy (Hc) and light (Lc) chains are indicated with an arrow. Polypeptides that are specifically immunoprecipitated with anti-GFP antibodies are indicated with asterisk.
(0.55 MB PDF)

Figure S5 Presence of subtilisin-resistant HDAC1 activity in the HIV-1 virus. Graphic representation of HDAC activity associated with HIV-1 virions, determined using a fluorimetric analysis. 3pV = three plasmid based vectors, and mock = culture supernatant of mock transfected cells. HIV-1 (3pV) and mock were treated with subtilisin and subjected to HDAC activity assay. Note the presence of subtilisin-resistant activity in HIV-1 (3pV), indicating that this activity is present within the virions.
(0.12 MB PDF)

Figure S6 Reduction in HDAC activity within the virions correlates with a reduction in infectivity of virus. Three different representative experiments are indicated to illustrate the correlation of virion-associated HDAC1 activity to infectivity. Viral supernatants collected from producer cells transfected with siHDAC1 or siControl from Experiments I–III were normalized for p24 and subjected to HDAC activity (A) and infectivity (B) assays. Bars represent the fold change in HDAC activity/ng p24 (A) and Infectivity, i.u./ng p24 when compared with virus produced in the presence of siControl (B).
(0.27 MB PDF)

Table S1 HDAC activity of virus produced in the presence of HDAC1H141A (activity/ng p24). 293T cells were co-transfected with three plasmid-based vectors along with either empty vector or vector expressing HDAC1H141A, and virions produced from these cells were used in HDAC activity assays. The results represent HDAC activity units minus background/ng p24.
(0.05 MB DOC)

Table S2 Gene symbols.
(0.03 MB DOC)
