# Supplementary figures and images for: Recruitment of a SAP18-HDAC1 Complex into HIV-1 Virions and Its Requirement for Viral Replication
Source: PLoS Pathog. 2009 Jun 5;5(6):e1000463. doi: 10.1371/journal.ppat.1000463 (PMC2685004; doi:10.1371/journal.ppat.1000463)

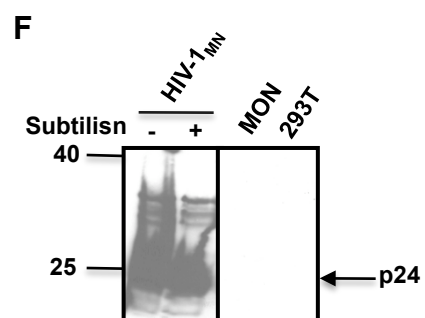

Supplement: Figure S1 — Immunoblot analysis of virions. Purified, concentrated, and subtilisin-treated HIV-1 mn virions were immunoblotted with various antibodies. The same blot was sequentially probed with α-INI1 (A), α-BRG1 (B), α-BAF170 (C), α-BAF155 (D), α-gp41 (E), and α-gp24 (F) antibodies, as indicated. Total protein lysates from MON (INI1−/− rhabdoid cells) and 293T (INI1+/+) were used as controls. (0.24 MB PDF) [file ppat.1000463.s001.pdf]

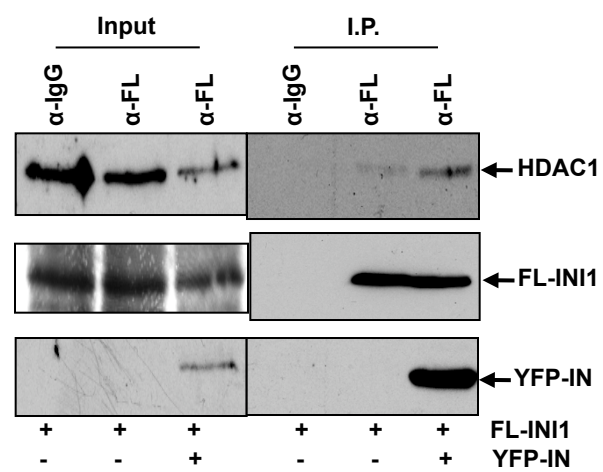

Supplement: Figure S2 — IN enhances the complex formation of INI1 with HDAC1. 293T cells were transfected with FLAG-INI1 and either pCDNA or YFP-IN plasmids and subjected to immunoprecipitation with either IgG or anti-FLAG monoclonal antibody agarose, as indicated. Immunoprecipitated complexes were analyzed by anti-FLAG monoclonal, anti-GFP polyclonal, and anti-HDAC1 polyclonal antibodies. Input lysates showing equal loading for FLAG-INI1, YFP-IN, and HDAC1 are shown. (0.14 MB PDF) [file ppat.1000463.s002.pdf]

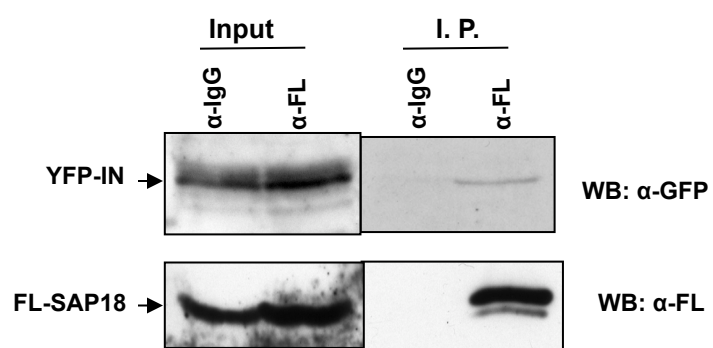

Supplement: Figure S3 — Interaction of IN with Sap18 in (INI1−/−) MON cells. MON cells were transfected with YFP-IN and FLAG-SAP18 plasmids and subjected to immunoprecipitation using either mouse IgG or anti-FLAG monoclonal antibody agarose. Immunoprecipitated complexes were analyzed using western blots with both anti-GFP polyclonal and anti-FLAG monoclonal antibodies. Input lysates showing equal loading for both YFP-IN and FLAG-SAP18 are shown. (0.15 MB PDF) [file ppat.1000463.s003.pdf]

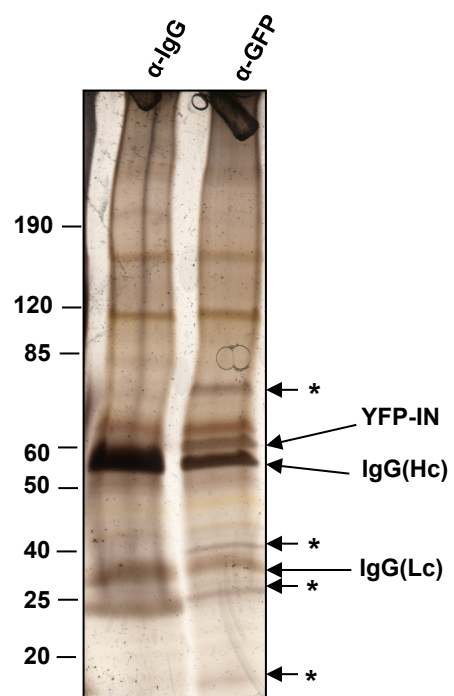

Supplement: Figure S4 — Silver stain analysis of immunoprecipitations to determine the specificity of interaction. Silver stain analysis of immunoprecipitations to determine the specificity of interaction. 293T cells were transfected with YFP-IN plasmid and subjected to immunoprecipitation using either mouse IgG or anti-GFP monoclonal antibody. Immunoprecipitated complexes were analyzed by SDS-PAGE followed by silver staining. Position of YFP-IN, IgG heavy (Hc) and light (Lc) chains are indicated with an arrow. Polypeptides that are specifically immunoprecipitated with anti-GFP antibodies are indicated with asterisk. (0.55 MB PDF) [file ppat.1000463.s004.pdf]

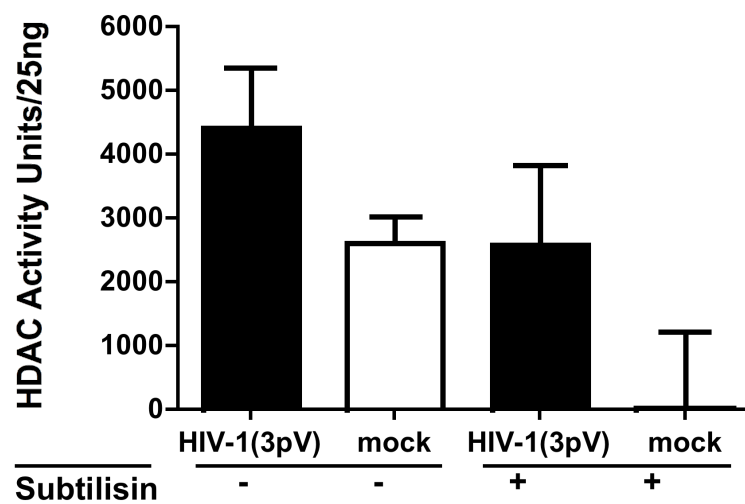

Supplement: Figure S5 — Presence of subtilisin-resistant HDAC1 activity in the HIV-1 virus. Graphic representation of HDAC activity associated with HIV-1 virions, determined using a fluorimetric analysis. 3pV = three plasmid based vectors, and mock = culture supernatant of mock transfected cells. HIV-1 (3pV) and mock were treated with subtilisin and subjected to HDAC activity assay. Note the presence of subtilisin-resistant activity in HIV-1 (3pV), indicating that this activity is present within the virions. (0.12 MB PDF) [file ppat.1000463.s005.pdf]

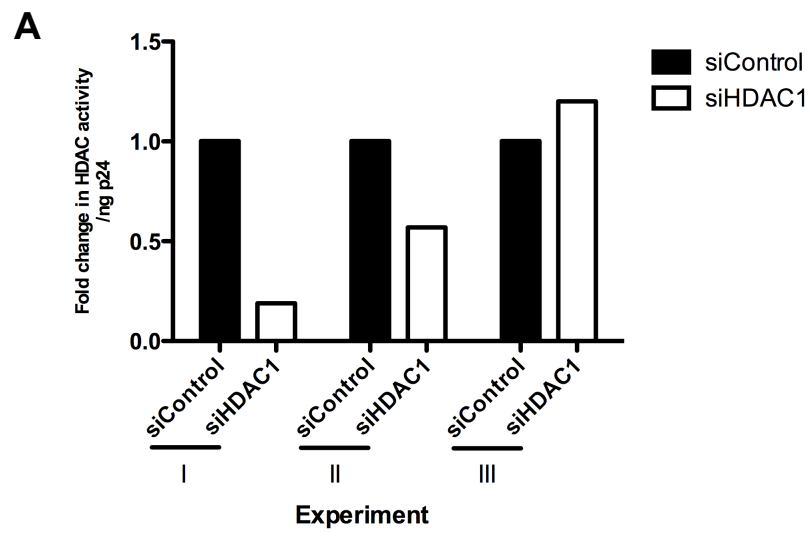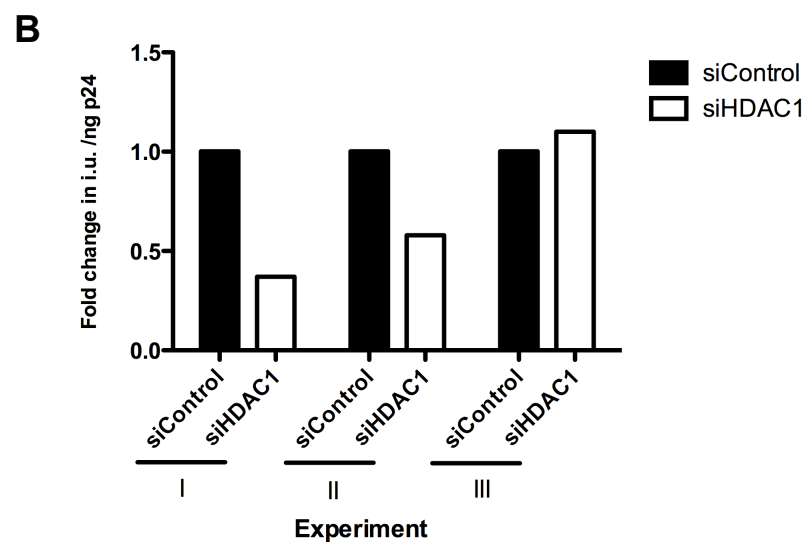

Supplement: Figure S6 — Reduction in HDAC activity within the virions correlates with a reduction in infectivity of virus. Three different representative experiments are indicated to illustrate the correlation of virion-associated HDAC1 activity to infectivity. Viral supernatants collected from producer cells transfected with siHDAC1 or siControl from Experiments I–III were normalized for p24 and subjected to HDAC activity (A) and infectivity (B) assays. Bars represent the fold change in HDAC activity/ng p24 (A) and Infectivity, i.u./ng p24 when compared with virus produced in the presence of siControl (B). (0.27 MB PDF) [file ppat.1000463.s006.pdf]
