# Supplementary material for: Recruitment of a SAP18-HDAC1 Complex into HIV-1 Virions and Its Requirement for Viral Replication
Source: PLoS Pathog. 2009 Jun 5;5(6):e1000463. doi: 10.1371/journal.ppat.1000463 (PMC2685004; doi:10.1371/journal.ppat.1000463)
Supplement: Table S1 — HDAC activity of virus produced in the presence of HDAC1H141A (activity/ng p24). 293T cells were co-transfected with three plasmid-based vectors along with either empty vector or vector expressing HDAC1H141A, and virions produced from these cells were used in HDAC activity assays. The results represent HDAC activity units minus background/ng p24. (0.05 MB DOC) [file ppat.1000463.s007.doc]

|  | (-) TSA | (+) TSA | (-) TSA | (+) TSA |
| --- | --- | --- | --- | --- |
|  | Empty vector | Empty vector | HDAC1^H141A^ | HDAC1^H141A^ |
| Experiment I |  |  |  |  |
|  | 4090.10 | 7.34 | 520.16 | 3.60 |
|  | 3697.58 | 85.26 | 335.76 | 201.21 |
|  | 3688.60 | 134.19 | 622.51 | 118.80 |
| **Average** | **3825.43** | **75.59** | **492.81** | **107.87** |
| Experiment II |  |  |  |  |
|  | 3120.36 | 267.79 | 775.36 | 243.93 |
|  | 3294.43 | 406.71 | 1025.86 | 452.14 |
|  | 4812.14 | 682.73 | 1555.02 | 1106.33 |
| **Average** | **3742.31** | **452.41** | **1118.74** | **600.80** |
| Experiment III |  |  |  |  |
|  | 1759.44 | N.D. | 748.44 | N.D. |
|  | 1790.24 | N.D. | 1162.96 | N.D. |
| **Average** | **1774.84** | **N.D.** | **955.70** | **N.D.** |
| Experiment IV |  |  |  |  |
|  | 580.54 | 223.14 | 238.26 | 35.16 |
|  | 370.10 | 0.00 | 239.66 | 0.00 |
| **Average** | **475.32** | **111.57** | **238.96** | **11.72** |
