# Supplementary material for: Recruitment of a SAP18-HDAC1 Complex into HIV-1 Virions and Its Requirement for Viral Replication
Source: PLoS Pathog. 2009 Jun 5;5(6):e1000463. doi: 10.1371/journal.ppat.1000463 (PMC2685004; doi:10.1371/journal.ppat.1000463)
Supplement: Table S2 — Gene symbols. (0.03 MB DOC) [file ppat.1000463.s008.doc]

| Gene Name/Symbol | Accession Number (NCBI) |
| --- | --- |
| INI1/SMARCB1/hSNF5/BAF47 | NM_003073 |
| BRG1/SMARCA4 | NM_003072.3 |
| BAF155/SMARCC1 | NM_003074 |
| BAF170 | NM_139067 |
| SAP18 | NM_005870 |
| SAP30 | NM_003864 |
| HDAC1 | NM_004964 |
| Sin3a | NM_015477 |
| HDAC3 | NM_003883 |
| CHD3 | N_001005271 |
| HIV-1 | NC_001802 |
